# Regional Population Forecast and Analysis Based on Machine Learning Strategy

**DOI:** 10.3390/e23060656

**Published:** 2021-05-24

**Authors:** Chian-Yue Wang, Shin-Jye Lee

**Affiliations:** 1 Graduate Institute of Urban Planning, National Taipei University, Taipei 237, Taiwan; andywang@mail.ntpu.edu.tw; 2 Institute of Management of Technology, National Chiao Tung University, Hsinchu 300, Taiwan

**Keywords:** population growth prediction, boosting regression

## Abstract

Regional population forecast and analysis is of essence to urban and regional planning, and a well-designed plan can effectively construct a sound national infrastructure and stabilize positive population growth. Traditionally, either urban or regional planning relies on the opinions of demographers in terms of how the population of a city or a region will grow. Multi-regional population forecast is currently possible, carried out mainly on the basis of the Interregional Cohort-Component model. While this model has its unique advantages, several demographic rates are determined based on the decisions made by primary planners. Hence, the only drawback for cohort-component type population forecasting is allowing the analyst to specify the demographic rates of the future, and it goes without saying that this tends to introduce a biased result in forecasting accuracy. To effectively avoid this problem, this work proposes a machine learning-based method to forecast multi-regional population growth objectively. Thus, this work, drawing upon the newly developed machine learning technology, attempts to analyze and forecast the population growth of major cities in Taiwan. By effectively using the advantage of the XGBoost algorithm, the evaluation of feature importance and the forecast of multi-regional population growth between the present and the near future can be observed objectively, and it can further provide an objective reference to the urban planning of regional population.

## 1. Introduction

Reliable regional population forecasting can provide important information for urban planning, especially for decision support in regional planning. Basically, the analysis of regional population forecasting can be applied to estimate the demand of land for residents, industries, public facilities, and so on. In general, the scale of population has determined the demand for land, public infrastructure, and urban services. Meanwhile, it also determines the demand for natural resources and hence may have a negative impact on the natural environment, and the development process in a densely populated area is sometimes restricted in accordance with the carrying capacity. Thus, how to accurately estimate population growth in the near future has become an issue for the pioneering work of urban planning. The widely applied methods for population forecasting include expert evaluations (e.g., Delphi method), stochastic population forecasts, cohort-component method, trend extrapolation, etc. [1]. However, most of the above-mentioned methods mainly focus on forecasting the population growth of a single region or country. As for the essence of conventional methods, it is difficult to either model or predict population growth across regions systematically. A recent contribution by [2] makes county population estimation with an interregional cohort-component model, and the multi-regional population forecasting is potentially possible under a well-specified cohort-component structure. In addition, the completeness of migration data of each county plays an essential role in the Isserman approach. However, the only drawback for the cohort-component type population forecasting is to allow the analyst to specify the demographic rates of the future, and it goes without saying that this tends to introduce a biased result in forecasting accuracy.

In the booming trend of artificial intelligence, a lot of novel machine learning methods have been applied to address practical problems in the real world, such as smart healthcare, smart manufacturing, and smart service, and this promotes quick development of machine learning. To effectively avoid the bias problem, this work proposes an innovative method based on machine learning to forecast multi-regional population growth. Firstly, it aims to discover hidden information between the city population and its potential population-related features, such as birth, death, and per capital income. To attain a reliable analysis, the city population is applied as the dependent variable to evaluate feature information gain toward target features, and which can be positively processed by several machine learning methods. Secondly, in addition to evaluating feature importance from the existing population database in the real world, the proposed work also tries to predict the variant of population feature importance in the near future. To achieve this purpose, machine learning methods are therefore considered in accordance with their good learning ability. Further, to reinforce the reliability of this work based on practical evidence, three inference models are applied in the comparison in Section 4—Simulation Experiment, including Linear Regression model (conventional method), LSTM model, and XGBoost Regression model. As for the recurrent neural network models of deep learning, designed for long-range effect for prediction, a long short-term memory network, also known as LSTM, is also applied for the comparison in this work. Basically, the above inference models are trained by the population data from the existing population database to predict the possible population in the near future, and the corresponding feature importance of predicted future population is then evaluated by the XGBoost algorithm again. Lastly, it extracts information gain toward each feature and then ranks the value with the whole-time range, which is presented in Section 5—Simulation Experiment.

## 2. Related Works

### 2.1. Essential Factors of Population Growth

The seminal paper of [3] argues that a consumer-voter (migrant) would choose a residential area which best satisfies citizen preference pattern for public goods and services, and this type of migration phenomenon is known as “voting with one’s feet”. The authors of [4] re-examined the hypothesis of Tiebout by drawing on interstate migration data of the United States over 1965–1970, and their empirical findings further consolidated Tiebout’s postulation that consumer-voter moves to the area where public goods are efficiently supplied to meet citizen needs. Moreover, successive studies with more elaborate model specification include [5,6,7], and generally these studies point out that local public expenditure is a key factor of influencing migration decision. Hence, the level of public expenditure might play an essential role in contributing to regional population growth through migration. Income per capita is another economic factor which might affect the population level of a region, and this variable reflects economic disparities among regions. Further, labour force tends to move from low-income regions to high-income regions, and empirical evidences from [8] show that GDP per capita is an important pull factor explaining the migration flow.

### 2.2. Deep Learning Application in Decision Support

Deep Learning is a rising field of Machine Learning, and it is popularly applied to various purposes of Artificial Intelligence. In addition, it often has amazing performance due to its deep neural-like structure. Due to the high performance of deep learning, applying deep neural networks (DNNs) to discover the hidden pattern has been considered a popular approach to study the complex data distribution. Furthermore, the methods corresponding to the boosting and bagging mechanism are another type of deep learning structure since each prediction tree is based on fitting the residual of the previous tree. For example, boosting and bagging trees are also capable of gaining a robust result by combining more than two base trees. The XGBoost algorithm was initially a research project conducted by Chen in 2016 [9], and has now become a popular research field in machine learning. As it applies residual error to build a boosting tree, the XGBoost algorithm has also been recognized as another form of a deep learning model. A series of works applying the XGBoost algorithm to address classification problems [10,11] or process prediction and estimation works [12,13,14,15], and further development of the algorithm is ongoing [16,17]. By examining feature importance, a lot of works based on XGBoost present good performance on finding interpretative information from information gain [13,18,19,20]. Thus, this work tries to combine the results of prediction with that of feature importance to observe the change between times, compared with feature ranking based on known and unknown data.

Long short-term memory was proposed by Jürgen Schmidhuber in 1997 [21,22]. In 1999 and 2000, Felix Gers designed the component “forget gate” in the structure of a recurrent neural network; it is a function of cell memory controlling the weight among layers in an LSTM model [23]. Afterwards, another famous recurrent residual network-based model, the gated recurrent unit (GRU), was introduced in 2014 [24]. In addition, Google and Facebook invested huge efforts in applying the LSTM model to process natural language processing (NLP) works [25,26]. Instead of applying ARIMA to forecast, the LSTM model is commonly used to predict time series data in half a decade [27]. Although the “transformer model” has replaced certain advantages of recurrent networks in the field of NLPs [28], RNNs still play an important role in processing time series forecasting works because of its intuitive mechanism to predict a timeframe step by step with memorization. In addition, the advantage of shrinking residuals in the last step makes boosting trees more effective in extracting high-impact features, which are already good at measuring feature by information gain in a leaf.

### 2.3. Potential Disadvantage of Conventional Models

With regards to time series forecasting, regression and autoregressive integrated moving average, also known as ARIMA, is a conventional method based on statistics. By observing data patterns, increasing high-performance function with coefficient or regressor to fit the data well can be regarded a good approach, but a regression often suffers from overfitting problems in complex coefficients and regressor settings. In addition, a simple regression model is not able to fit a non-visible data well. Although the lasso regression or L2 penalty term might reduce the risk of overfitting, the interpretations of constrain coefficients are sacrificed. In addition, more disadvantages of applying regression models to process medical data have been raised in [29]. As for the ARIMA model, there are a series of necessary processes to define whether the data is suitable for application, such as seasonality and stationarity. In terms of using multiple variables on the ARIMA model, it is rare to only apply ARIMA to process multivariable data, although integrating ARIMA with multivariable regression to address different problems is applicable [30]. Therefore, ARIMA still mainly focuses on calculating univariables to predict future values in the assumption of retaining the same mean, variance, and log pattern. However, this hypothesis makes it hard for ARIMA to handle unexpected incidents as it is against assumptions or is exempted from pre-processing. Furthermore, it has been mentioned that applying ARIMA requires more technical knowledge because of mathematical sophistication in theory [31].

## 3. Boosting Regression-Based Method and Recurrent Neural Network

Firstly, the mechanism of the boosting method and the process of the XGBoost algorithm are described. In addition, the information gain of examining feature importance toward the target features is described. Furthermore, the prediction model, LSTM, is described with typical equations in Section 4.

### 3.1. Gradient Boosting-Based Method

In processing continuous data toward population growth, the mechanism designed to approximate the residual between the observed and predicted values is ideal for processing continuous data in this work. Since it is the algorithm, instead of the analysts, that decide how important a feature is, it is more objective. The basic loss function of calculating the residual is presented as follows:

(1) $$l\left(y_{i},\;{\hat{y}}_{i}\right)={\left(y_{i}-{\hat{y}}_{i}\right)}^{2}$$

where $y_{i}$ is the observed value, ${\hat{y}}_{i}$ is the predicted value, and i the index of the data set.

As for the gradient boosting algorithm, the corresponding loss function is presented in the following equation:

(2) $$l\left(y_{i},\;{\hat{y}}_{i}\right)=\frac{1}{2}{\left(y_{i}-{\hat{y}}_{i}\right)}^{2}$$

where $y_{i}$ is the observed value, ${\hat{y}}_{i}$ is the predicted value, and i is the index of the data.

Based on Equations (1) and (2), the effect of derivation, 1/2, takes advantage of decreasing the algorithm complexity by averaging the residual summary in the initial and last step of the entire procedure of the XGBoost algorithm.

### 3.2. XGBoost Algorithm

The mechanism of the XGBoost algorithm is designed as a boosting-based algorithm. With preference to build a stump at each round and then figure up the residual for the prediction, the mechanism of the XGBoost algorithm brings in a medium-sized tree comprising of leave restriction and normalization for the purpose of avoiding the problems of high variation and overfitting. Further, the procedure of the XGBoost algorithm is presented by the Algorithm 1:
**Algorithm 1**. XGBoost algorithmInput:     Data ${\left\{\left(x_{i},\;y_{i}\right)\right\}}_{i=1}^{n}$, and a differentiable Loss Function, as the algorithm (1): $l\left(y_{i},\;{\hat{y}}_{i}=F\left(x\right)\right)=\frac{1}{2}{\left(y_{i}-{\hat{y}}_{i}\right)}^{2}$
  Step 1:       Initialize model with a constant value: $F_{0}\left(x\right)=argmin\sum_{i=1}^{n}L\left(y_{i},\;r\right)$
  Step 2:       for m = 1 to M:(1)Calculate $r_{im}=-{\left[\frac{\partial L\left(y_{i},{\hat{y}}_{i}\right)}{\partial {\hat{y}}_{i}}\right]}_{F\left(x\right)=F_{m-1}\left(x\right)}$ for i=1…n(2)Fit a regression tree to the $r_{im}$ values and build terminal regions $R_{jm},$ for $j=1\ldots J_{m}$(3)For $j=1\ldots J_{m}$ complete $\gamma_{jm}=argmin\sum_{x_{i}\in R_{ij}}L\left(y_{i},\;F_{m-1}\left(x_{i}\right)+\gamma \right)$(4)$F_{m}\left(x\right)=F_{m-1}\left(x\right)+v\sum_{j=1}^{J_{m}}r_{jm}I\left(x\in R_{jm}\right)$  Step 3:       Output $F_{M}\left(x\right)$
 where i represents the index of data, n  is the total number of data, γ certainly refers to the average of observed data, m means the $m_{th}$ tree, M is the total amount of tree, j means the $j_{th}$ residual in the $m_{th}$ tree, and v means the learning rate or the distance of moving step toward the gradient of residual.

The algorithm focuses on reducing the difference between the observed and predicted values by continually optimizing the loss function, and the output can potentially prevent the overfitting problem, because of its advantage—restricting the gradient with learning rate.

(3) $$l^{\left(t\right)}=\overset{T}{\underset{j=1}{\sum }}\left[G_{j}w_{j}+\frac{1}{2}\left(H_{j}+\lambda \right)w_{j}^{2}\right]+\gamma T$$

where *l* means the number of leaves, $G_{j}$ = $\sum_{i=1}^{n}g_{i}$, $w_{j}=f_{t}\left(x_{i}\right)$,$\;H_{j}=\sum_{i\in I_{j}}h_{i}$, $h_{i}=\partial^{2}{}_{{\hat{y}}^{\left(t-1\right)}}l\left(y_{i},{\hat{y}}^{\left(t-1\right)}\right)$, λ is the Lagrange multiplier penalizing the L2 norm to prevent the overfitting problem, $w_{i}\;$ represents the score on the j-th leaf, γ means the number of leaves, and T is the number of nodes.

### 3.3. Gain

As for the functionality, Gain is recognized as the advantage of performing a prediction capability to either fit or separate data, and the novel tree-based classification algorithm, classification, and regression tree (CART), works based on Gain mechanism. Moreover, the algorithm of gain can be presented as follows:

(4) $$Gain=\frac{1}{2}\left[\frac{G_{L}^{2}}{H_{L}+\lambda }+\frac{G_{L}^{2}}{H_{R}+\lambda }-\frac{{\left(G_{L}+G_{R}\right)}^{2}}{H_{L}+H_{L}+\lambda }\right]-\gamma$$

where $G_{L}^{2}={\left(\sum_{i\in I_{L}}g_{i}\right)}^{2}$, $G_{R}^{2}={\left(\sum_{i\in I_{R}}g_{i}\right)}^{2}$,$\;H_{L}=\sum_{i\in I_{L}}h_{i}$, and $H_{R}=\sum_{i\in I_{R}}h_{i}$. Moreover, $g_{i}=\partial_{{\hat{y}}^{\left(t-1\right)}}l\left(y_{i},{\hat{y}}^{\left(t-1\right)}\right)$ and $h_{i}=\partial^{2}{}_{{\hat{y}}^{\left(t-1\right)}}l\left(y_{i},{\hat{y}}^{\left(t-1\right)}\right)$ are first and second order gradient statistics on the loss function, respectively.

The default value of γ is initially set at 0. Once the gain is presented as a negative value, the algorithm removes the branch. Moreover, once the gain of the root with two leaves is presented as a negative value, the algorithm removes the root. In other words, it therefore implies that the whole tree is abandoned, and the original value will be taken as the prediction in this step in the output. Thus, this is called “pruned” [32].

### 3.4. XGBoost Regression Model

The XGBoost Regression model is a tree-like boosting-based algorithm, like the XGBoost model, except for the fact that the XGBoost Regression model uses Similarity Score of the related split points to calculate Information Gain. Further, the equation for Similarity Score is:

(5) $$S=\frac{\sum_{k=1}^{n_{\alpha }}\alpha^{2}}{n_{\alpha }+\lambda }$$

where *S* is the Similarity Score, α is the residual of each datapoint in the split point, $n_{\alpha }$ is the number of the residuals in the split point, and λ is the regularization parameter.

### 3.5. Long Short-Term Memory Network

Long short-term memory is an exception of recurrent neural networks, and it is tasked with several challenges, including translation, classification, and time series forecasting. In time series forecasting, the design of a memory cell can relieve the problem of gradient vanishing, which is a norm in recurrent network models considering long-time range data. In this case, an integration effect comprising memory cell, input gate, output gate, and forget gate can capsulate the previous information into the next LSTM neuron. Since its first introduction in 1995, there has been a series of variant LSTMs. However, a typical LSTM network usually consists of an input gate, an output gate, and a forget gate; it has been applied in this work. Furthermore, compact equations for the forward LSTM network can be presented as the following:

(6) $$F_{t}=\sigma_{S}\left(W_{F}x_{t}+U_{F}h_{t-1}+b_{F}\right)$$

(7) $$I_{t}=\sigma_{S}\left(W_{I}x_{t}+U_{I}h_{t-1}+b_{I}\right)$$

(8) $$O_{t}=\sigma_{S}\left(W_{O}x_{t}+U_{O}h_{t-1}+b_{O}\right)$$

(9) $${\tilde{C}}_{t}=\sigma_{T}\left(W_{C}x_{t}+U_{C}h_{t-1}+b_{C}\right)$$

(10) $$C_{t}=F_{t}⊙C_{t-1}+I_{t}⊙{\tilde{C}}_{t}$$

(11) $$H_{t}=O_{t}⊙\sigma_{R}\left(C_{t}\right)$$

where $x_{t}\in {\mathbb{R}}^{d}$ is the input vector, $F_{t}\in {\mathbb{R}}^{h}$ is the output vector of forget gate, $I_{t}\in {\mathbb{R}}^{h}$ is the output vector of input gate, $O_{t}\in {\mathbb{R}}^{h}$ is the output vector of output gate, $H_{t}\in {\mathbb{R}}^{h}$ is the output vector, ${\tilde{C}}_{t}\in {\mathbb{R}}^{h}$ is the output vector of memory cell, $C_{t}\in {\mathbb{R}}^{h}$ is the output vector of current cell, $W\in {\mathbb{R}}^{h\times d}$ is the weight metric, $U\in {\mathbb{R}}^{h\times h}$ is the recurrent connection metric, $b\in {\mathbb{R}}^{h\times h}$ is the bias metric, d is referred as t×features, t is the time step, S is the Sigmoid function, and R is the PReLU function.

## 4. Simulation Experiment

### 4.1. Data Description

In this work, population data was adopted from the Directorate-General of Budget, Accounting and Statistics, Executive Yuan, R.O.C. (Taiwan). It is open data managed by the Taiwan Government and can be found at: https://www.ris.gov.tw/app/portal/346 (accessed on 1 January 2021). The data has six features, including birth population, death population, net immigration population, per capital income, city annual expend, and total population. Among these features, total population was selected as the target dependent output variable to examine feature importance. The time range was from 2009 to 2018, which is a decade data. In addition, the data is extracted yearly, so there are 10 data for each city. In total, there are 60 data for six cities, including New Taipei city, Taipei city, Taoyuan city, Taichung city, Tainan city, and Kaohsiung city. According to Section 3, three methods were applied in the simulation experiments: Linear Regression model, LSTM model, and XGBoost Regression model; the result of each model is compared based on MAPE. Mean absolute percentage error (MAPE) is the relationship between the model loss and the real value in percentage, and is widely used to evaluate the model. Furthermore, the MAPE equation is presented as follows:

(12) $$\mathrm{MAPE}=\frac{1}{n}\overset{n}{\underset{i=1}{\sum }}\left|\frac{\hat{y_{i}}-y_{i}}{y_{i}}\right|$$

where *n* is the sample size, $y_{i}$ is the actual value, and $\hat{y_{i}}$ is the forecast value. When zero exists in the actual value, to avoid calculation errors, instead of MAPE, the MAE equation is used to evaluate the model.

Furthermore, the reason for using MAPE over MAE is because the latter tends to ignore the meaning of sample mean. For example, it might be acceptable to have MAE = 10 in a sample data with mean = 10,000, but in the case of mean = 0.001, it is obviously unacceptable. In this work, the mean is different from city to city, so using MAPE for rescaling each center of error into the same standard can make the comparison clear—the index range is set from 0 to 100.

### 4.2. Experiment Design

In accordance with the general modelling validation of machine learning, the original data is usually divided into two sets: training data and validation data. However, as the available population data is only from 2009 to 2018, the amount of data is somewhat insufficient for model training. To overcome this technical difficulty, this simulation applies the “sliding window” to complete the modelling, and training accuracy is measured by MAPE, compared to real historical data. Meanwhile, this simulation experiment focuses on finding changes in the essential features that could affect population growth in the near future. Therefore, the experiment is divided into two parts: in the first phase, it applies the XGBoost model to fit the data and then applies Gain to rank the feature towards the total population. In the second phase, three typical models, the XGBoost Regression model, the Linear Regression model, and the LSTM model, are applied to predict the data for the near future with regards to total population and the rest of the features until 2025. Based on these data formats, the data is fit and then predict until 2025. Later, the feature importance ranking evaluates the priority of feature importance from 2009 to 2025, processed by the Linear Regression model, the LSTM model, and the XGBoost Regression model, toward the total population with the same time range. Thus, this simulation experiment further observes the ranking difference of feature importance between known data and predicts data. Thus, the primary essence of this simulation includes:

- The MAPE is applied as the measuring criteria to evaluate modelling performance in the comparison, as shown in Table 1. By observing a fitting tendency between the real historical data and the forecasted data from 2009 to 2018, it can further confirm the reliability of the forecast results from 2019 to 2025.
- Three inference models are applied in the comparison in this work, including the Linear Regression model (conventional method), the LSTM model, and the XGBoost Regression model. In addition, the comparisons are summarized in Table 1.

**Figure 1:**
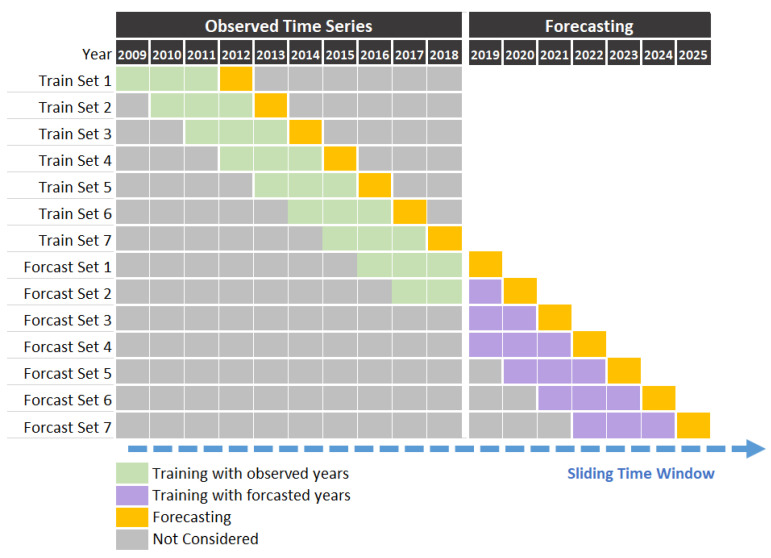
Demonstration of Sliding Windows to Forecast Every Year with the Previous 3 Years.

Among different time ranges of training data within six cities, the MAPEs of Linear Regression, LSTM, and XGBoost Regression were best in a time range of 5, 3, and 5 years, as shown in Table 1. In the training procedure, the time window of each model contains its best time range of input data plus one time range output for the prediction. On the ground of the sliding window, the n-y behind each model means it contains n years in a window. For example, 3 y indicates prediction for 2012 by considering data for the previous three years—2009, 2010, and 2011. In the simulation, the six variables of 2009–2011, including birth, death, immigration, city annual expend, per capital income, and population, are defined as the input of the training set to predict the population of 2012. As shown in Figure 1, there are seven training sets from 2009–2018 in this simulation based on a sliding window, and the performance is measured with MAPE. For each model, the year range of the sliding window that performs best is then selected as the time range of the model in the comparison.

### 4.3. Near Future Forecasting with Linear Regression, XGBoost Regression, and LSTM Models

**Figure 2:**
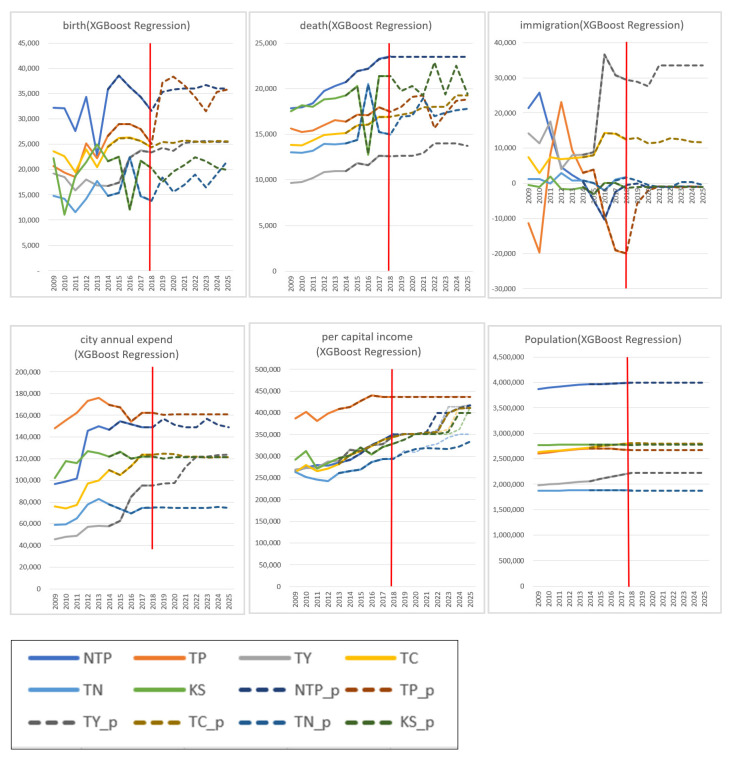
Population forecasting by XGBoost Regression for the near future. Whilst solid lines stand for the real-world data, the dashed lines stand for the forecasted results, where the six cities are presented in 12 different colours. NTP is New Taipei, TP is Taipei, TY is Taoyuan, TC is Taichung, TN is Tainan, and KS is Kaohsiung city.

In order to evaluate the change in feature importance ranking from 2019 to 2025, the prediction of each feature to 2025 is required. As described before, the time window contains the best year range and the last value of each model for calculating the MAPE between observed values and predict values. In addition, the time windows sliding rightward one-column-step after each data value is established. As a result, a complete time range with 10 data is split into six windows toward the inference models. Furthermore, near future forecasting toward each feature of the primary inference model—XGBoost Regression—based on its best time range is illustrated in Figure 2.

As shown in Table 1 and Figure 2, the prediction performance of the XGBoost Regression model is the most effective among all the inference models in population forecasting as a whole, as the fitting tendency between the real-world data and the forecasted data is much more closed than the other inference models in the comparisons, especially by viewing average MAPE. Moreover, the prediction performance of “immigration” can be effectively recognized, implying that the XGBoost Regression model has the potential to possess high consistency with certain hyperparameters.

Moreover, the red belt represented the boundary between the known data and the predicted data. In other words, data located on the left are the real-world data, and data on the right are the forecasted data generated by the inference models. The real-world data are presented with solid lines and the forecasted data with dashed lines. Real-world data and forecasted data both appear in the year range of 2012–2018 and accuracy is measured by MAPE for each model. The MAPE of each feature and the average MAPE among different model-year-ranges were found to be best in the time range of three years, five years, and five years for the Linear Regression model, the LSTM model, and the XGBoost Regression model, respectively. Further, the prediction performance of the XGBoost Regression model was found to be the most effective in population forecasting.

### 4.4. Feature Importance in the Present, across a Known Time to the Near Future

**Figure 3:**
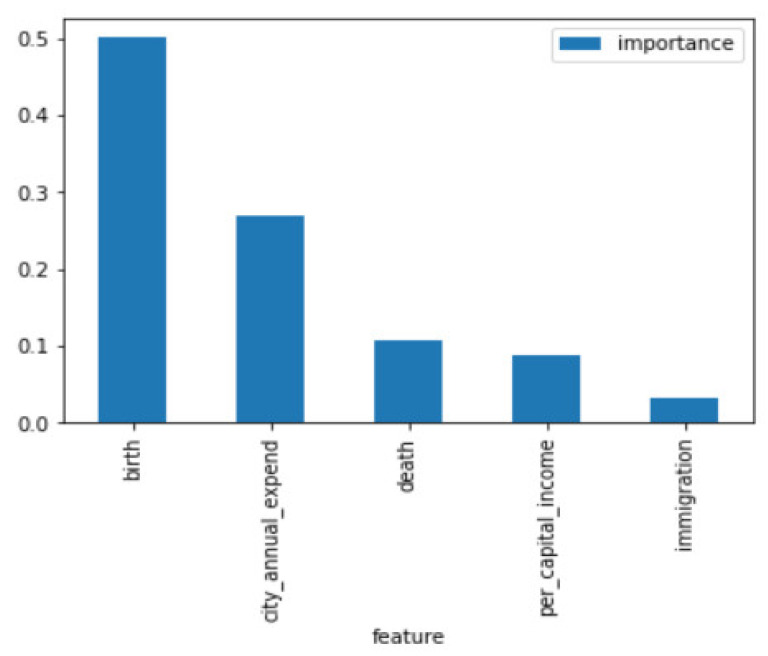
Feature Importance in accordance with known data; the data year range is 2009–2018.

**Figure 4:**
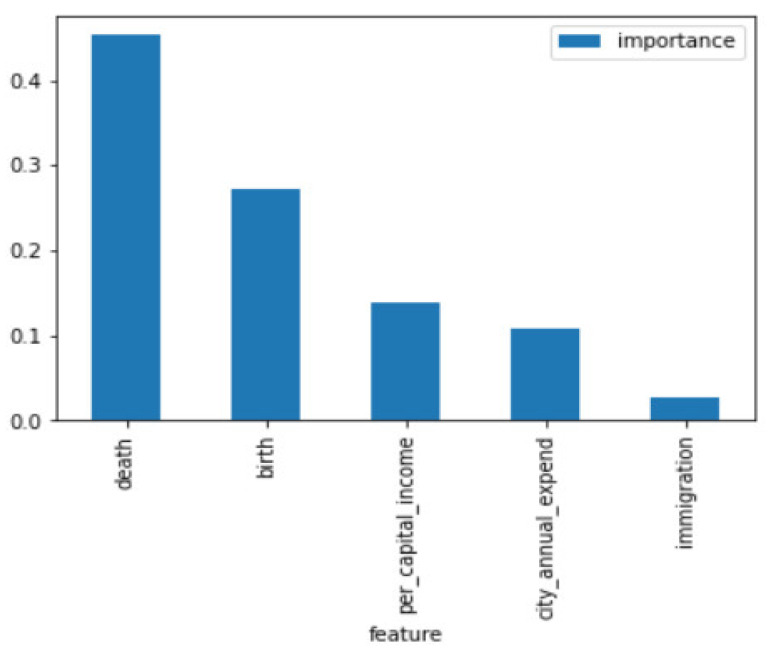
Feature Importance in accordance with both known and unknown data; the data year range is 2009–2025.

As described in the previous section, the first part examines the feature importance ranking for the total population across the cities. The feature importance ranking is shown in Figure 3 and Figure 4.

In Figure 3, the values of the features are information gain, based on Equation (4). The simulation results of the XGBoost Regression model help recognize the important features affecting population growth in the six cities; the features recognized for six major cities in Taiwan are tabulated in Figure 4. As observed, “birth” possesses the highest feature importance among the known data (data year range 2009–2018), and “death” possesses the highest feature importance among the known and unknown data (data year range 2009–2025). In accordance with the simulation results, the features with the highest feature importance are different among the known data and the forecasted data, but “immigration” has the lowest feature importance in both known data and forecasted data, as shown in Figure 3 and Figure 4.

## 5. Conclusions

Regional population forecast and analysis is of essence in urban and regional planning, and well-designed planning can effectively construct a sound national infrastructure and stabilize a positive population growth. Traditionally, either urban or regional planning relies on the opinions of demographers in terms of how the population of a city or a region will grow. Multi-regional population forecast is currently possible, carried out mainly on the basis of the Interregional Cohort-Component model. While this model has unique advantages, several demographic rates are determined by the decisions made by primary planners. Hence, the only drawback in cohort-component type population forecasting is that it allows the analyst to specify the demographic rates of the future, and it goes without saying that this tends to introduce a biased result in forecasting accuracy. To avoid this problem, this work proposes a machine learning-based method to objectively forecast multi-regional population growth. Thus, this work, drawing upon newly developed machine learning technology, attempts to analyze and forecast the population growth of major cities in Taiwan. By effectively using the XGBoost algorithm, the evaluation of feature importance and forecast of multi-regional population growth in the present and the near future can be objectively observed, and can further provide an objective reference for urban and regional planning.

## Figures and Tables

**Table 1:** The MAPE of each feature and the average MAPE among different model-year-ranges in the Linear Regression model, the LSTM model, and the XGBoost Regression model were best in a time range of three years, five years, and five years, respectively. As a whole, the XGBoost Regression model outperformed the other two.

	Feature	Birth	City Annual	Death	Immigration	Income	Population	Average MAPE
Models in Different Year Range								
**Linear_Regression_3Y**		0.30265	0.36806	0.24133	6.35127	0.17148	0.23123	1.27767
**Linear_Regression_4Y**		0.36432	0.39890	0.26973	26.03689	0.18115	0.26782	4.58647
**Linear_Regression_5Y**		0.34876	0.37034	0.28464	11.57862	0.15104	0.25222	2.16427
**LSTM_3Y**		1.40973	1.47480	1.43107	10.09746	0.29646	1.31306	2.67043
**LSTM_4Y**		1.34646	1.48777	1.42434	11.45912	0.28670	1.30690	2.88521
**LSTM_5Y**		1.21405	1.27438	1.41467	13.70877	0.27888	1.30739	3.19969
**XGBoost_3Y**		0.01310	0.00396	0.00210	0.42950	0.00149	0.00017	0.07505
**XGBoost_4Y**		0.00725	0.00179	0.00101	0.11286	0.00080	0.00012	0.02064
**XGBoost_5Y**		0.00201	0.00069	0.00062	0.13376	0.00047	0.00009	0.02294

## Data Availability

The data is managed by the Taiwan Government and which can be found at: https://www.ris.gov.tw/app/portal/346 (accessed on 1 January 2021).
